# Intrinsically Elastic Organic Semiconductors (IEOSs)

**DOI:** 10.3390/molecules26206130

**Published:** 2021-10-11

**Authors:** Fei Liu, Xueling Hou, Benlin Hu, Runwei Li

**Affiliations:** 1 Institute of Materials Science, School of Materials Science and Engineering, Shanghai University, Shanghai 200072, China; liufei2021@nimte.ac.cn; 2 CAS Key Laboratory of Magnetic Materials and Devices and Zhejiang Province Key Laboratory of Magnetic Materials and Application Technology, Ningbo Institute of Materials Technology and Engineering, Chinese Academy of Sciences, Ningbo 315201, China

**Keywords:** elastic organic semiconductors, elastic semiconductors, intrinsically elastic semiconductors, field-effect transistors (FETs), block copolymers, conjugated polymers

## Abstract

Elastic semiconductors are becoming more and more important to the development of flexible wearable electronic devices, which can be prepared by structural engineering design, blending, and the intrinsic elastification of organic semiconductors (intrinsically elastic organic semiconductor, IEOS). Compared with the elastic semiconductors prepared by structural engineering and blending, the IEOS prepared by organic synthesis has attracted numerous attentions for its solution processability and highly tunable chemical structures. For IEOSs, reasonable designs of synthetic routes and methods are the basis for realizing good mechanical and electrical properties. This brief review begins with a concise introduction of elastic semiconductors, then follows with several synthetic methods of IEOSs, and concludes the characteristics of each method, which provides guidance for the synthesis of IEOSs in the future. Furthermore, the properties of IEOSs are involved from the aspects of electrical, mechanical properties, and the applications of the IEOSs in elastic electronic devices. Finally, the challenge and an outlook which IEOSs are facing are presented in conclusion.

## 1. Introduction

Since polyacetylene, a conductive polymer material with semiconductor properties, was reported by Heeger in 1977 [1], organic semiconductors have entered into a rapid development stage [2]. Organic semiconductors are conjugated molecules based on a π- bond. Compared with inorganic semiconductors, organic semiconductors can be structure-adjustable, solution-processable, intrinsically flexible, and compatible with flexible substrates, and therefore they have attracted widespread attention [3]. In terms of molecular structure, organic semiconductors can be roughly divided into organic small molecules and polymers. The organic semiconducting small molecules are clear in chemical structure, easy to purify, and are then able to be used in the preparation organic semiconductor single crystals with high purity; whereas conjugated polymers have a certain molecular weight distribution and easy modification on chemical structures, and also can be easy to prepare films by solution processing [4,5]. The devices based on organic semiconductors, such as organic field-effect transistors (OFETs) [6], organic photovoltaics (OPVs) [7], and organic light-emitting diodes (OLEDs) [8], have made great strides in the past decades.

Flexibility and elasticity are considered to meet the development trend of electronic equipment along with the intelligentization of human life, such as enabling expandable and fold-able smartphones, epidermal medical devices, and wearable electronic devices [9,10,11]. Obviously, semiconductors are an important component of electronic devices, and, therefore, the flexible and elastic semiconductors are irreplaceable roles in future electronics. Conjugated polymer semiconductors have shown important potential applications in wearable electronic devices and electronic skin due to their intrinsic flexibility [12,13,14]. However, the strain resistance of conjugated polymers is still too small (generally less than 5%) to meet the strain requirements of wearable devices (20–50%) [15]. Therefore, elastic semiconductor appears timely. There are three main ways to manufacture elastic semiconductors: structural engineering design, blending, and molecular engineering.

**Figure 1:**
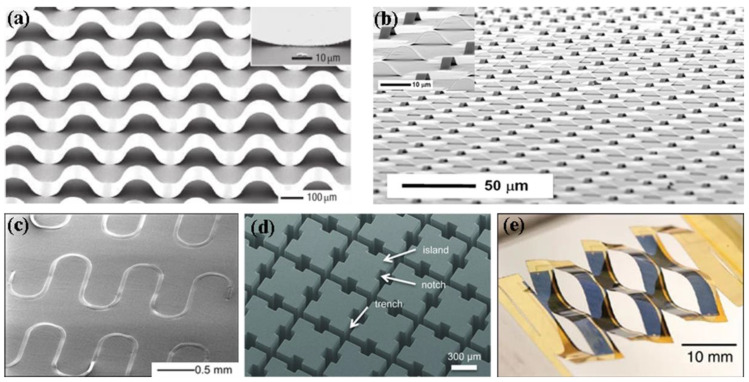
Structural engineering design model drawing: (**a**) buckling [20], (**b**) mesh [30], (**c**) serpentine [18], (**d**) islands [16], and (**e**) kirigami [31].

**Figure 2:**
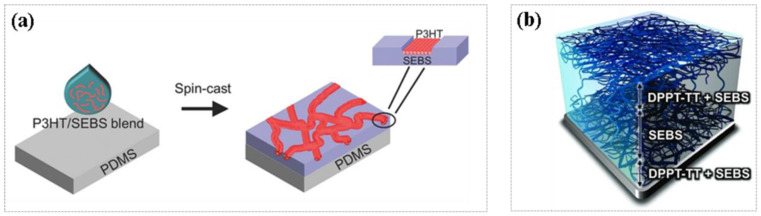
(**a**) Schematic diagram of P3HT and SEBS mixed in solution (**left**); distribution of P3HT and SEBS blend on PDMS substrate (**right**), the insert showed P3HT nanofibrils indented in the rubber surface [21]. (**b**) Microscopic 3D schematic of DPPT-TT and SEBS blending [22].

Structural engineering design refers to connecting rigid semiconductor device units with elastic structures that can withstand strain, such as islands [16], wrinkles, or serpentines [17,18,19] (Figure 1), so as to realize the overall elastification of devices. Sun and coworkers reported buckled GaAs nanoribbons in 3D space and then embedded them in PDMS. The electrical properties of the materials obtained by this method were almost unchanged under 100% strain [20]. Although the devices prepared by engineering design have good mechanical and perfect electrical properties, the complicated fabrication process and high cost in device miniaturization limit the application of engineering design. The blending method is related to the physical blending of organic semiconductors and elastomers to prepare elastic electronic devices. The elastic semiconductors obtained by blending have both the electrical properties of rigid semiconducting materials and the elasticity of elastomers. As shown in Figure 2a, poly(3-hexylthiophene) (P3HT) was blended with styrene-ethylene/butylene-styrene (SEBS) block copolymer elastomers to obtain elastic devices with hole mobility of about 2 × 10^−3^ cm^2^ V^−1^ s^−1^ at 50% strain [21]. An elastic semiconducting film with hole mobility higher than 1 cm^2^ V^−1^ s^−1^ under 100% strain was obtained by blending DPP-based conjugated polymer (DPP-TT) with elastomer SEBS (Figure 2b) [22]. The advantages of this method are a low preparation cost and simple process; however, there are interface problems and poor fatigue resistance after long-term use.

Molecular engineering involves the synthesis of IEOSs by covalently linking conjugated units and elastic units in the same molecular chain. At the molecular level, this ensures that IEOSs possess both charge transport and elasticity, simultaneously. IEOSs are single substances at the molecular level and have the designability of chemical structures. At the same time, both the whole and each part of IEOSs can withstand large strain, which can be compatible with the existing preparation technology for organic semiconductor devices and easily realize the miniaturization of devices. The IEOSs are the focus in the development trend of elastic semiconductors.

In this review, we only attempt to give a brief introduction to the molecular engineering to synthesize elastic semiconductors. If the readers are interested in other preparation methods and processing of elastic semiconductors, some related well-written papers can be referred [21,22,23,24,25,26,27,28,29]. This paper mainly deals with the synthesis methods, properties (electrical and mechanical properties), and potential applications of intrinsic organic elastic semiconductors in the past years. Finally, the above-mentioned contents are summarized, and a reasonable prospect for the development and the potential application of IEOSs are presented to end our discussion.

## 2. Synthetic Methods

**Scheme 1:**
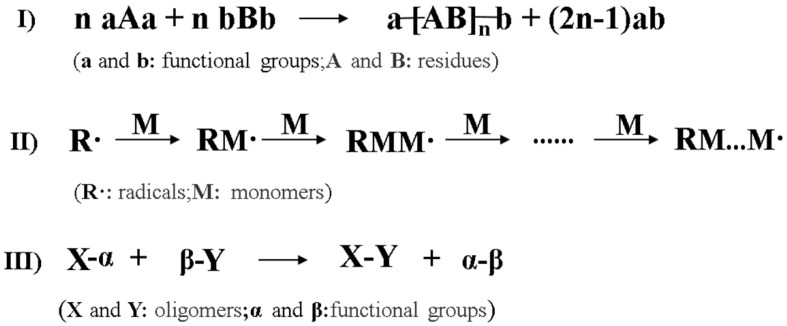
Reaction general formulas to synthesize IEOSs, (**I**) coupling and polycondensation, (**II**) free radical polymerization, and (**III**) click reaction.

The aggregation structures of IEOSs in solid states are similar to those in other elastomers. The charge transport capacity of IEOSs originates from the continuous phase composed of conjugate moieties, while the deformability under stress comes from the other continuous phase formed by the soft segments. Therefore, the synthesis of conjugate parts and soft parts, as well as the connection between the two parts, should be considered together synergistically. At present, the synthetic methods of IEOSs reported in the literature mainly include coupling polymerization, polycondensation, free radical polymerization, and click reaction polymerization, etc. The general formulas of these reactions are listed in Scheme 1.

### 2.1. Coupling

**Figure 3:**
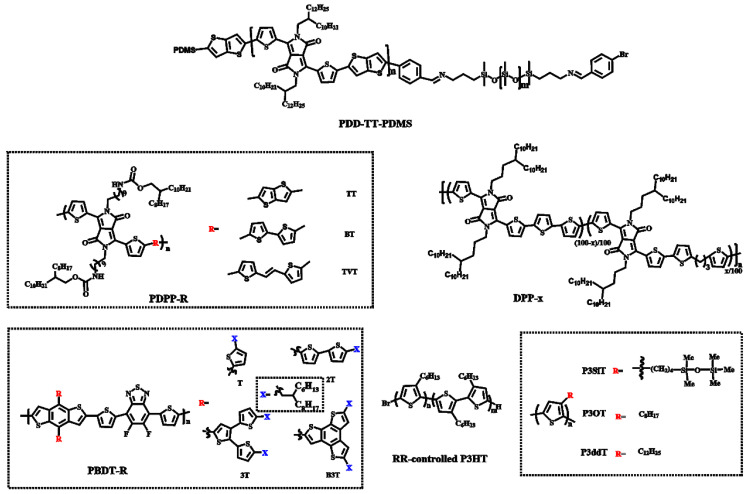
Chemical structures of IEOSs obtained by coupling reaction.

Coupling reaction has good universality and is frequently used in conjugated polymer synthesis [32]. The chemical structures of some reported IEOSs synthesized by coupling reactions are shown in Figure 3. Diketopyrrolopyrrole (DPP) is a typical building block for organic semiconductors. PDPP-TT-PDMS triblock copolymer was synthesized through Stille coupling [33]. PDPP-TT (BT, TVT) and DPP-X polymers are also synthesized by Stille coupling by inserting conjugation-break spacers into the DPP-based polymer backbones [34,35,36]. P3HT is another classical building block in polymer semiconductors. Kim [37], Son [38], and Park [39] synthesized P3HT based IEOSs with different regioregularity (RR) through Grignard metathesis (GRIM) by Kumada coupling; Shen [40] and Smith [41] synthesized P3SiT, P3OT, and P3ddT by changing side chains by Kumada coupling. Benzodithiophene (BDT)-based polymers (PBDT-T, PBDT-2T, PBDT-3T, and PBDT-B3T) with different side chains was prepared by Suzuki coupling [42]. However, coupling reaction is difficult to scale up due to harsh reaction conditions, cumbersome separation and purification of products, and low utilization rate of raw materials.

### 2.2. Polycondensation

**Figure 4:**
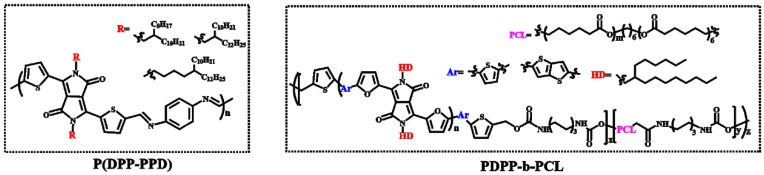
Chemical structure of IEOSs obtained by polycondensation reaction.

Polycondensation is a kind of reaction in which functional groups repeatedly condense to form polymers while small molecules are split out. Unlike coupling reaction, polycondensation reaction has poor adaptability because it requires the participation of functional groups prone to chemical reactions. Generally, it does not need catalysts, is not sensitive to water and oxygen, and the product is usually easy to purify; therefore, it is frequently employed in industrial production [43]. In the process of synthesizing IEOSs, it is often necessary to connect the two ends of the conjugated monomers or oligomers with functional groups that can perform condensation reactions. For example, Tran introduced aldehyde groups into the DPP structure to form DPP-CHO, which was used with polycondensation with amino groups to form P(DPP-PPD) (Figure 4) [44]. A degradable IEOS block copolymer (PDPP-b-PCL) was synthesized by introducing the PCL oligomers in the polymer backbone [45].

### 2.3. Free Radical Polymerization

**Figure 5:**
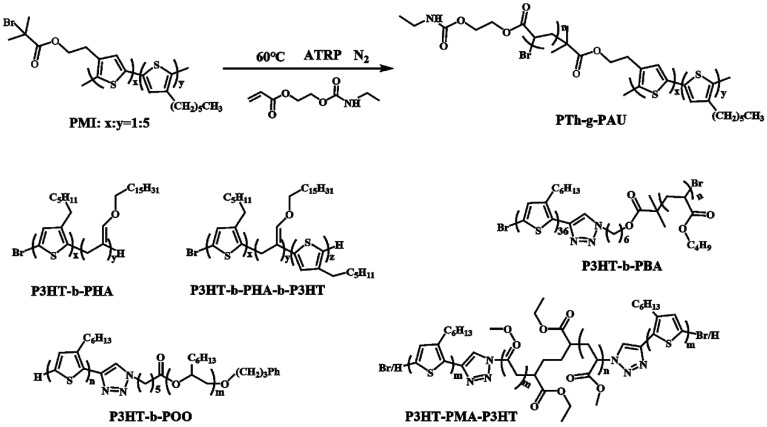
IEOSs obtained by free radical polymerization and click reaction.

Free radical polymerization is a kind of reaction in which monomers are activated into active free radicals by light, heat, radiation, and initiator, and then polymers are formed by chain polymerization. It plays an extremely important role in polymer chemistry and has been widely used for massive polymer production in industry [46]. As shown in Figure 5, three series of graft copolymers PTh-g-PAU were synthesized by atom transfer radical polymerization (ATRP) using a P3HT copolymer macroinitiator (PMI) [47]. GRIM and ATRP were combined to create a “one-pot” method to synthesize a series of P3HT-b-PHA-b-P3HT triblock and p3HT-b-PHA diblock copolymers [48].

### 2.4. Click Reaction

Click reaction is often used to connect oligomers, and has attracted extensive attention due to its advantages of simple reaction conditions, fast reaction rate, and easy product processing. Poly (butyl acrylate) (PBA) oligomer was synthesized by ATRP and P3HT oligomer was synthesized by GRIM, respectively, and then the two oligomers were connected into P3HT-b-PBA polymer by click reaction [36]; P3HT-PMA-P3HT triblock and P3HT-b-POO polymers were synthesized by the click method [49,50], respectively (Figure 5). However, the click reaction requires high activation energy, and the azide intermediates are susceptible to causing safety accidents [51].

## 3. Fundamental Properties

Elastic semiconductor materials are generally evaluated by mechanical and electrical properties. Among them, strength, elongation (or crack onset strain), and mobilities are the most important evaluation indexes. Therefore, the following contents are mainly summarized from the mechanical properties and mobilities of intrinsically elastic semiconductors reported in the literature.

### 3.1. Mechanical Properties

**Figure 6:**
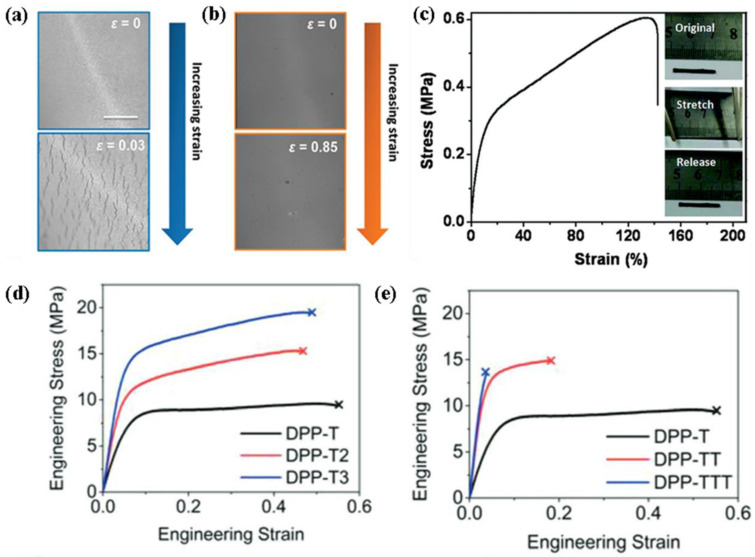
Microscopy images of strained PDPP-TT (**a**) and PDPP-TT–PDMS (**b**), respectively (scale bars denote 50 µm and ε is the amount of applied strain). (**c**) The strain–stress profile of P3HT–PMA–P3HT triblock copolymer, illustrated by optical photographs at different tensile strains. Stress-strain curves of (**d**) DPP polymer (DPP-T, DPP-T2, and DPP-T3) films with different thiophene unit numbers and (**e**) DPP polymer (DPP-T, DPP-TT, and DPP-TTT) films thickened with thiophene rings in different sizes.

There are many factors affecting the mechanical properties of polymer materials, such as the unit structures in polymer backbones, side chains, polymer molecular weight, and so on. Therefore, we can tune the above-mentioned factors to obtain suitable mechanical properties. Usually, the amounts of each batch of synthesized semiconductors (tens to hundreds mg) are not enough for mechanical tests (usually >1g), therefore the crack onset strains are employed to evaluate the stretchability of the IEOSs. For example, cracks appeared in PDPP-TT films when the strain reached 3%, while Ditte and coworkers inserted elastomer PDMS with Mn = 25 kg mol^−1^ into the main chain of PDPP-TT, and no cracks remained after the strain reached 85% [33] (Figure 6a,b). The crack onset strain of P3HT block copolymer can exceed 100% by introducing elastic block copolymer [49] (Figure 6c). In DPP-based block copolymers, the modulus, strength, and elongation at the break of elastic semiconductors were modulated by changing the amount and size of inserted thiophene [52] (Figure 6d,e). The crystallinity of the material can be adjusted by changing RR, thus affecting its mechanical properties. Tensile modulus of P3HT can be reduced from 287 MPa (RR = 98%) to 13 MPa (RR = 64%) [37]. In addition, Zheng et al. reduced the crystallinity of the polymer by inserting H-bonding conjugation breaker into polymer backbones, thus increasing the elasticity of the polymer [53].

**Figure 7:**
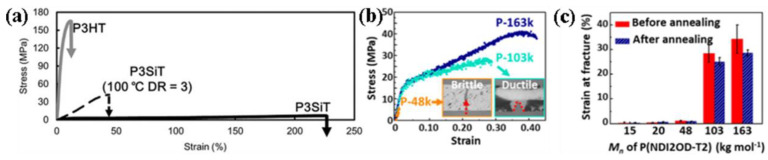
(**a**) Stress–strain curves of P3SiT and P3HT under three tensile cycles at 100 °C. (**b**) stress–strain curves and (**c**) elastic moduli of polymers (p (NDI2OD-T2)) with different molecular weights.

The extension of polymer side chains increases the number of physical crosslinking points and the effect of entanglement between chains, thus increasing the elasticity of the materials. In addition, side chains increase the plasticity of the material, which also enhances the elasticity. P3SiT was synthesized by introducing disiloxane moieties into P3HT, and it was found that the fracture strain of P3SiT was more than 200%, much higher than that of P3HT (14%) [40] (Figure 7a). Isoindigo-bithiophene-conjugated copolymers (PII2T) were obtained by incorporating octyldecyl (OD) and polyacrylate amide (PAAm) in the side chain. The H-bonds (especially on the sidechain) and the branched side chains increase the interaction between the polymer chains, and the crack onset strain increases and the elastic modulus decreases compared with the original polymer films [54].

The mechanical properties can also be affected by the molecular weight of polymers [55]. For example, as shown in Figure 7b, the mechanical properties of polymers PDPP-TT were affected by PDMS with different molecular weights [33]. On the other hand, different degrees of polymerization of polymer will affect its mechanical properties. The fracture strain of P(NDI2OD-T2) increased 26-fold when the molecular weight increased from 48 kg mol^−1^ to 103 kg mol^−1^ (Figure 7c) [56].

### 3.2. Mobility

Mobility (μ), an average drift velocity of carriers per unit electric field intensity, represents the performance of semiconductor materials, including electron mobility and hole mobility. The mobility of organic semiconductors can usually be increased by tuning their structural units or increasing their crystallinity. For elastic semiconductors, both mechanical properties and mobilities should be considered synchronously. Some recent representative work about high mobility in the field of IEOSs will be discussed.

**Figure 8:**
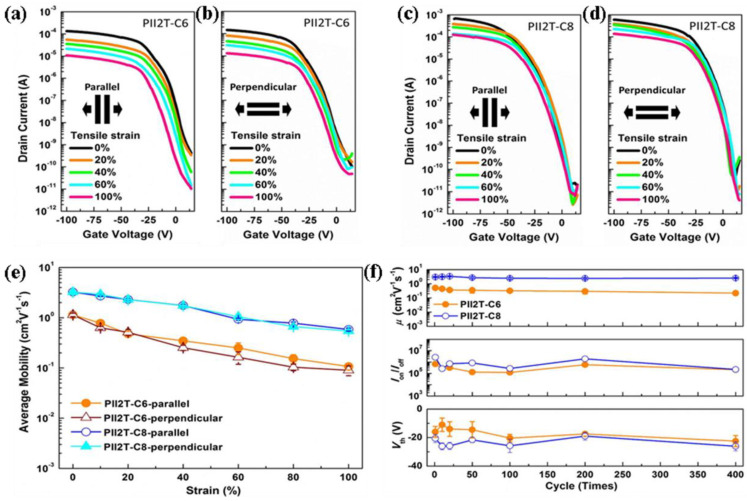
Transfer characteristic curves of polymer (PII2T-C6, PII2T-C8) films in parallel (**a**,**c**) and perpendicular (**b**,**d**) direction of charge transfer under different strains. (**e**) Field-effect mobility of polymer (PII2T-C6, PII2T-C8) films in horizontal and vertical directions under different strain levels. (**f**) Mobilities, on/off ratios, and threshold voltages of polymer (PII2T-C6, PII2T-C8) films during 400 tensile/release cycles at 60% strain.

The hole mobility of the PDPP-TT-PDMS mentioned above was 0.1 cm^2^ V^−1^ s^−1^ without deformation, and remained almost unchanged after 1500 cycles under 50% tensile strain [33]. Transfer characteristic curves of conjugated polymers (PII2T-C6, PII2T-C8) films based on isoindigo (II) have similar changes in horizontal and vertical directions under different strains. The initial hole mobility of PII2T-C8 film was 3.24 cm^2^ V^−1^ s^−1^. After 400 cycles of 60% strain, it was still 1 cm^2^ V^−1^ s^−1^, which is obviously better than that of PII2T-C6 film. This could be attributed to the better ductility and denser molecular packing structure of PII2T-C8. In addition, two other electrical properties, on/off current ratio (I_on_/I_off_, the ratio of the on-state and off-state current under specific gate voltage (Vg)) and threshold voltage (V_th_, the gate voltage at which the device starts to turn on), remained unchanged after the film cyclic strain [57] (Figure 8). P3HT-conjugated polymer films have low mobility, for example, the hole mobility of P3HT (RR = 98) film was 0.181 cm^2^ V^−1^ s^−1^ [37]. The hole mobility of block polymers such as P3HT-b-PHA-b-P3HT and P3HT-b-PBA films are lower than 0.06 cm^2^ V^−^^1^ s^−^^1^ [36,48]. In BDT-based polymers prepared by Huang, PBDT-3T film had a hole mobility of 0.30 cm^2^ V^−1^ s^−1^ under 60% tension [58]. In Tian’s works, PBIBDF-BT thin films exhibited bipolar transport properties with both electron and hole mobility greater than 0.1 cm^2^ V^−1^ s^−1^ at 100% strain [42].

## 4. Applications

IEOSs are mainly used in stretchable electronic devices, such as organic elastic field- effect transistors (OEFETs), elastic sensors, and elastic resistive memory devices. The successful preparations of these elastic electronic devices can obviously build a solid foundation for the good development of wearable electronic devices, electronic skins, and biological monitoring.

The mobilities of IEOSs are usually characterized by OEFETs. For example, the OEFET prepared with DPP-PDCA polymer film still maintains the device mobility of 1.2 cm^2^ V^−1^ s^−1^ under 100% strain [59]. Sensors based on the OFETs have been used to detect gas, temperature, pH, light, and pressure [60]. For example, the pressure sensor based on PIDT-BT had a sensitivity of 452.7 KPa^−1^ [61]. A gas sensor based on P-channel (DPPT-TT) and N-channel (P(NDI2OD-T2)) was reported, and it was found that the DPPT-TT polymer having a thickness of around 2 nm showed a sensitivity toward ammonia of about 80%, and the device also had significant sensing responses toward ethylene and ethanol vapors [62]. An optical response device using DPP-based polymer (PDPP-DBTE) showed an open state light response of about 2.5 AW^−1^ [63]. Elastic semiconductors are also necessary for elastic resistive memory devices. The resistive memory devices prepared by P3HT-b-POO-b-P3HT block copolymer film as a resistive layer exhibited non-volatile flash characteristics. The device can withstand up to 80% strain and the switch hardly changes after 500 stretches under 50% strain [50]. With the development of IEOSs, we believe more applications in the field of stretchable electronic devices will be reported.

## 5. Conclusions and Perspectives

Elastic semiconductors have gradually become an essential part of stretchable electronic devices. Intrinsic organic elastic semiconductors have attracted much attention. In this review, we summarized the recently reported fabrication methods of elastic semiconductors, and gave some detailed introductions to the synthesis, properties, and applications of IEOSs.

The development of IEOSs is based on the progress of organic semiconductors. DPP, P3HT, and isoindigo-based polymers have been reported and synthesized by coupling, polycondensation, free radical polymerization, and click reaction. When the elasticity is realized by changing the side chain and the molecular weight of the polymer, the mobility decreases due to its reduced crystallinity; whereas the elasticity obtained by inserting soft blocks in the polymer backbones can effectively avoid the above problems. At present, the precise aggregation structures of polymers is not easily regulated to form an ideal “conductive pathway” in the elastic semiconductors. As a result, the properties (mechanical properties, electrical properties, and so on) of materials cannot be predicted accurately. This requires us to have a deeper understanding of the aggregation structure of polymers and its regulation methods. As we mentioned above, the use of block copolymers with soft segments and hard segments to form a typical elastomer should be an ideal and effective way towards high performance IEOSs, in which a bicontinuous phase will be formed. The soft phase can take the stress and be deformed, while the hard phase will keep continuous and transport charge carriers effectively.

Presently, the applications of IEOSs are still mainly in FETs. On one hand, IEOSs cannot maintain high mobility after repeated stretching and compression cycles; on the other hand, the current works are mainly focused on P-type IEOSs, and a rarity of works on N-type and bipolar IEOSs have been reported, which greatly limits the development of logic circuits, light-emitting field effect transistors, and other organic electronic devices. With the concerted efforts of researchers in the fields of physics, chemistry, materials, and devices, we have every reason to believe that IEOSs will attract increasing attention and be applied in a widely range of wearable devices.

## Data Availability

Not applicalbe.
